# Canal wall down versus canal wall up surgeries in the treatment of middle ear cholesteatoma

**DOI:** 10.3906/sag-1904-109

**Published:** 2019-10-24

**Authors:** Recep KARAMERT, Fakih Cihat ERAVCI, Süleyman CEBECİ, Mehmet DÜZLÜ, Mehmet Ekrem ZORLU, Nagihan GÜLHAN, Hakan TUTAR, Mehmet Birol UĞUR, Ayşe İRİZ, Yıldırım Ahmet BAYAZIT

**Affiliations:** 1 Department of Otolaryngology, Faculty of Medicine, Gazi University, Ankara Turkey; 2 Department of Otolaryngology, Faculty of Medicine, Medipol University, İstanbul Turkey

**Keywords:** Cholesteatoma, mastoidectomy, tympanoplasty, ossicular replacement

## Abstract

**Background/aim:**

To compare outcomes of canal wall up (CWU) and canal wall down (CWD) techniques in the treatment of middle ear cholesteatoma.

**Materials and methods:**

Medical records of 76 patients who had a primary surgery due to middle ear cholesteatoma between July 2015 and November 2017 were reviewed retrospectively. Hearing thresholds, speech discrimination scores (SDS), recurrences, and revision surgeries of CWU and CWD surgeries were compared.

**Results:**

Of 76 cholesteatoma cases, 40 (52.6%) had a CWU and 36 (47.4%) had a CWD operation. Postoperatively, the mean air conduction thresholds were significantly better in CWU compared to CWD surgeries (P = 0.016). The presence of the stapes and the type of reconstruction material used did not have a significant effect on auditory success rates (P = 0.342 and P = 0.905, respectively). Auditory success was affected by the status of the middle ear mucosa as well. The recurrence and revision rates did not differ between the surgical techniques (P > 0.05).

**Conclusion:**

Status of the middle ear mucosa and external auditory canal are important factors affecting the outcomes in cholesteatoma. Instead of a CWD surgery, a CWU surgery seems applicable in cases of cholesteatoma when the bone in the external auditory canal is not eroded by the disease.

## 1. Introduction

Cholesteatoma is a cystic lesion composed of keratinized squamous epithelium [1]. 

Once diagnosed, surgical treatment is inevitable because of its progressive destructive character and potential to cause functional loss and severe complications [2]. The aim of cholesteatoma surgery is to completely eradicate the disease and to obtain satisfactory hearing.

Canal wall down (CWD) and canal wall up (CWU) are the surgical techniques used in the treatment of cholesteatoma. These techniques are mainly distinguished by preservation of the external ear canal. CWD is considered to be the more effective method for eradication of cholesteatoma, as it allows the evaluation of mastoid and middle ear structures with a wide angle of view. However, a self-cleaning cavity cannot usually be obtained in the CWD technique and the patient should avoid contact with water, which will lead to social limitations. These problems are avoided in the CWU technique because the anatomy is preserved. However, residual disease and recurrences may be more common in CWU than in CWD [3]. In addition, hearing results with CWU are considered to be better than with CWD [4–7].

After CWU surgeries, the inability to observe the cavity directly during postoperative otoscopic examination is an important handicap for the follow-up of cholesteatoma recurrence. Hence, a second-look surgery for residual disease monitoring is recommended [8].

Nonecho–planar diffusion-weighted magnetic resonance imaging (non-EPI-DW MRI) is also favored as an alternative method in postoperative cholesteatoma screening after CWU surgeries [9,10].

Sinus tympani and facial recesses are the most common localizations from which cholesteatoma recurrence originates [11,12]. These areas are difficult to visualize in the CWU technique through the ear canal or the mastoid cavity. A posterior tympanotomy (PT) provides direct access to the facial recesses and sinus tympani and helps to eradicate cholesteatoma in the CWU technique [13].

The aim of this study was to compare hearing outcomes and disease eradication rates for CWU and CWD techniques in the treatment of middle ear cholesteatoma. Improvements in hearing thresholds and speech discrimination scores (SDS), rates of recurrence, and revision surgeries were the main parameters compared between CWU and CWD surgeries. Factors influencing hearing outcomes and disease eradication were further evaluated.

## 2. Materials and methods

Medical records of 76 patients who had a primary surgery due to acquired middle ear cholesteatoma between July 2015 and November 2017 were reviewed retrospectively, and the data related to preoperative and postoperative hearing thresholds, speech discrimination scores (SDS), surgical techniques, recurrences, and revision surgeries were recorded after ethical committee approval was obtained from the university. Informed consent was obtained from all individual participants. There were no patients with congenital, petrous bone, or revision cholesteatoma.

The surgeries were performed under general anesthesia, using a retroauricular approach. A CWD surgery was performed in cases of erosion in the bone of the external auditory canal. A CWU surgery was performed when the bone in the external auditory canal was intact. The CWU surgeries were performed with a PT (CWU+PT) or without a PT (CWU–PT) depending upon accessibility to the cholesteatoma matrix in the facial recess and sinus tympani. In CWU, the incus and head of the malleus were removed as needed, and the posterior buttress was removed when a PT was performed. Surgeries were classified according to the criteria determined by the International Otology Outcome Group and the International Consensus on the Categorization of Tympanomastoid Surgery, in order to comply with the current nomenclature [14]. In this context, CWU–PT, CWU+PT, and CWD surgeries performed in this study match with M1a, M1b, and M2c surgeries in the current nomenclature, respectively.

In the postoperative period, all patients were followed up with periodic otoscopic examinations for recurrence. Instead of a second-look surgery for recurrent or residual cholesteatoma, the CWU cases (M1a and M1b) were assessed with non-EPI-DW MRI at least 1 year after the surgery.

Epitympanic cholesteatoma was accepted as recurrence and cholesteatoma behind the mesotympanum was encountered as residual disease, as suggested by Jackler et al. [15]. The term recidivism was used to define both recurrence and residual disease [3,16].

The hearing outcomes were reported in accordance with the Committee on Hearing and Equilibrium Guidelines of the American Academy of Otolaryngology - Head and Neck Surgery, and comply with Level 1 guidelines [17]. A scattergram was formed for the techniques as described in the new standardized format of the guidelines [18]. The last performed postoperative audiometry after the first year of each case was obtained, and 10 dB gain in air conduction or air-bone gap of 20 dB or less in postoperative audiometry was accepted as an auditory success.

## 3. Results

The SPSS 20.0 software program (IBM Corp., Armonk, NY, USA) was used for the statistical analyses. The Mann–Whitney and chi-squared tests were used to compare quantitative and ordinal variables, respectively. P-values less than 0.05 were considered statistically significant.

There were 42 (55.3%) males and 34 (44.7%) females with a mean age of 33.8 (min: 6, max: 61) years. The disease was on the left side in 34 (44.7%) and on the right side in 42 (55.3%) patients. Mean follow-up time was 25.1 months (min: 12, max: 54 months). 

Of 76 cholesteatoma cases, 40 (52.6%) had a CWU and 36 (47.4%) had a CWD operation. The demographic features did not differ between these groups (P > 0.05). In CWU operations, a PT was also performed (M1b) in 28 (36.8%), while a PT was not performed (M1a) in 12 (15.8%) patients. 

Overall, preoperative and postoperative mean pure tone air conduction thresholds were 45.9 dB and 40.03 dB, respectively (P = 0.003). The mean air–bone gap changed from 31.4 dB to 24.8 dB after the operation (P < 0.001). Preoperative and postoperative mean SDS were 91.9% and 91.37%, respectively (P = 0.564).

**Figure 1:**
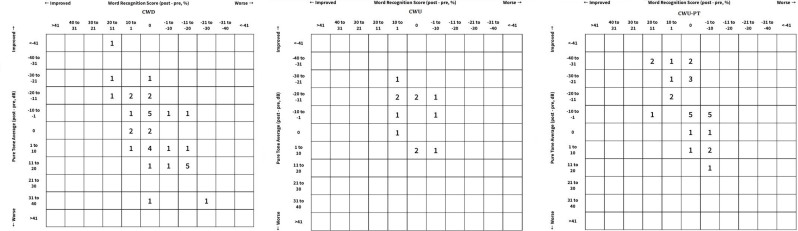
Scattergram diagrams of each surgical technique.

Preoperatively, the mean pure tone air conduction thresholds were 42.6 dB, 46.4 dB, and 46.7 dB in M1a, M1b, and M2c groups, respectively, which were not significantly different (P = 0.759). Postoperatively, the mean air conduction thresholds were 34 dB, 34.25 dB, and 46.53 dB in M1a, M1b, and M2c groups, respectively, which was significantly better in CWU compared to CWD surgeries (P = 0.016) (Figure 1).

**Table 1:** Auditory success and failure rates of each hearing reconstruction technique.

	Auditory success		Auditory fail	
	N	%	N	%
Autograft	34	60.7	22	39.3
TORP	7	58.3	5	41.7
PORP	3	75.0	1	25.0
Bone cement	1	100.0	0	0.0
N/A	2	66.7	1	33.3

**Figure 2:**
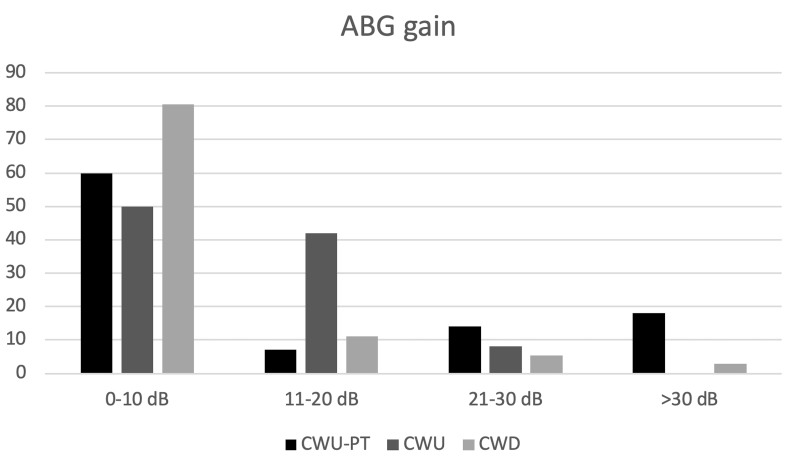
Comparison of ABG gains between surgical techniques.

Postoperatively, air–bone gap gains were 10.2 dB, 12.3 dB, and 0.8 dB in M1a, M1b, and M2c surgeries, respectively, which was significantly better in CWU compared to CWD surgeries (P = 0.006). There was no significant difference between M1a and M1b surgeries in terms of mean air–bone gap gains (P = 0.542) (Figure 2). No statistical significance was found between the surgeries regarding the changes in SDS (P = 0.417).

**Table 2:** Comparison of hearing parameters according to presence of the stapes.

	Stapes	N	Mean	Std. deviation	Mean difference	*P-value
Pre-op PTA	+	45	43.62	17.76	–5.67	0.152
	-	31	49.29	15.22		
Post-op PTA	+	45	39.11	20.88	–2.24	0.616
	-	31	41.35	16.03		
Gain (dB)	+	45	4.51	15.12	–3.36	0.391
	-	31	7.87	18.74		
Pre-op gap	+	45	29.67	11.94	–4.33	0.163
	-	31	34.00	14.81		
Post-op gap	+	45	23.04	15.14	–4.28	0.217
	-	31	27.32	14.07		
ABG gain	+	45	6.40	13.22	–.28	0.938
	-	31	6.68	17.93		
Pre-op SD	+	45	91.96	11.79	–.01	0.996
	-	31	91.97	9.43		
Post-op SD	+	45	90.44	13.01	–2.27	0.396
	-	31	92.71	8.42		
SD gain	+	45	–1.56	7.91	–2.30	0.213
	-	31	0.74	7.74		

*Independent samples test.

The stapes was intact in 45 (59%) cases. There was stapes superstructure erosion requiring total ossicular chain reconstruction in 31 (41%) patients. Otografts (incus, malleus, or cortical bone) were used for ossicular reconstruction in 56 (73.6%) patients. Hydroxyapatyte total ossicular reconstruction prosthesis (TORP) and partial ossicular reconstruction prosthesis (PORP) were used in 12 (15.7%) and 4 (5.2%) patients, respectively. In one (1.3%) patient, incudostapedial bridging was made with bone cement, and the ossicular chain was left intact in 3 (3.9%) patients. There was no statistically significant difference between CWD and CWU surgeries by the means of reconstruction material used (P = 0.483). The gains in air–bone gap and SDS were not related with the reconstruction material used (P = 0.999 and P = 0.819, respectively) (Table 1). The presence of stapes did not influence the gains in air–bone gap closure or SDS (P > 0.05) (Table 2). Presence of the stapes and the type of reconstruction material did not have a significant effect on auditory success rates (P = 0.342 and P = 0.905, respectively).

A hyperplastic middle ear mucosa with granulation tissues was present in 57 (75%) of the patients. In 19 (25%) patients, the middle ear mucosa was normal in appearance. The gains in air–bone gap closure and SDS were significantly better in the presence of healthy middle ear mucosa (P = 0.015 and P = 0.01, respectively). Overall, auditory success was achieved in 47 (61.8%) patients. The rates of auditory success were 75% and 47.2% in CWU and CWD surgeries, which were significantly higher in the CWU group (75%) (P = 0.045). Auditory success was affected by the status of the middle ear mucosa as well. The rates of auditory success were 84.2% and 54.4% in the presence of a normal and hyperplastic middle ear mucosa, respectively (P = 0.028). 

The rates of cholesteatoma recidivism which required revision surgery were 8.3%, 10.7%, and 19.4% for M1a, M1b, and M2c groups, respectively (Table 3). The cause of recidivism was residual disease in all 7 M2c and 1 M1a groups. In the M1b group, 1 of 3 recidivism cases was due to a secondary retraction pocket formation; this case was accepted as a recurrence. In the remaining 2 cases, the residual disease was found behind the mesotympanum. An additional 4 patients in the M1b group underwent revision surgery due to retraction pocket development without a cholesteatoma formation. Overall revision surgery rates were 8.3%, 25%, and 19.4% for the M1a, M1b, and M2c groups, respectively. The recurrence and revision rates did not differ between the groups (P > 0.05). In all cases of revision, the middle ear mucosa was found out to be hyperplastic in the initial surgery. A revision surgery was not required for any of the patients who had a normal middle ear mucosa in the initial surgery. There was a significant relationship between the status of the middle ear mucosa and revision surgery (P = 0.016).

**Table 3:** Comparison of cholesteatoma recidivism between surgical techniques.

		Recidivisim		Total
		+	-	
M1a	N	1	11	12	%	8.3	91.7	100.0
	M1b	N	3	25	28	%	10.7	89.3	100.0
M2c		N	7	29	36	%	19.4	80.6	100.0
	Total	N	11	65	76	%	14.5	85.5	100.0

## 4. Discussion

The present study demonstrated better hearing outcomes and similar recurrence rates for CWU surgeries when compared with the CWD technique in cases of cholesteatoma when the bone in the external auditory canal was not eroded by the disease. The status of the middle ear mucosa and external auditory canal was found to be the most important factor affecting the outcomes in cholesteatoma surgery.

The primary objective of cholesteatoma surgery is to eradicate squamous epithelium from the middle ear and mastoid cavity. However, maintaining satisfactory hearing and prevention of recurrences are also important.

CWD techniques are favored because of the better visualization of the middle ear status compared to CWU techniques [19]. Better visualization allows for complete eradication of the disease from the middle ear. The vast majority of the studies in the literature report a lower recurrence rate in CWD surgeries when compared with the CWU technique [3,20–22] By contrast, in the present series, cholesteatoma recurrence and revision surgery rates were similar between the CWD and CWU techniques. However, the relatively short follow-up duration (around 2 years) might have an impact on that similarity, because a 5-year follow-up is advised to make an accurate recurrence evaluation [3].

The traditional follow-up method for residual or recurrent cholesteatoma after CWU surgeries is a second-look operation [8]. However, non-EPI–DW MRI has emerged within the last decade as a noninvasive, less time-consuming, and cost-effective alternative to second-look operations in postoperative cholesteatoma screening after CWU surgeries. Cholesteatomas show distinct signals in non-EPI–DW MRI and are distinguished from postoperative mucosal changes successfully [9,10]. De Foer et al. showed high sensitivity, specificity, and positive and negative predictive values with non-EPI–DW MRI in residual cholesteatoma screening after CWU surgeries. They claimed non-EPI–DW MRI is capable of detecting even very small cholesteatomas and has the ability to select appropriate candidates for second-look surgery, avoiding unnecessary surgery [9]. In the present series, we also used non-EPI–DW MRI for cholesteatoma screening after CWU surgeries. None of the patients in which a high-signal intensity lesion was not present in non-EPI–DW MRI has undergone a revision surgery.

In general, extension or severity of the disease can affect the preference of surgical technique in cases of cholesteatoma. In addition, status of the middle ear mucosa is an important factor affecting postoperative outcomes [23–25]. The present study also supports this contention since the mucosal status significantly correlated with revisions, recurrences, and hearing outcomes, regardless of the surgical technique applied.

Ossiculoplasty is critical in hearing restoration. In CWU surgeries, PT is considered to help visualize the position of ossiculoplasty material. However, that maneuver usually does not affect hearing outcomes [26]. In our study, although the best hearing outcome could be achieved with the M1b technique, there was no statistically significant difference between M1a and M1b surgeries. The hearing results of CWU surgeries were better than those of CWD surgeries. These results suggest that hearing results are related to preservation of the external auditory canal. 

Stapes superstructure is considered to play an important role in hearing restoration, and better hearing results with PORP compared to TORP have been reported by some authors [5,27,28], while other authors did not find a significant effect of the stapes superstructure on postoperative hearing outcome [25,29,30]. In the present study, the presence of the stapes superstructure did not influence hearing outcomes. We think that a well-aerated middle ear cleft with a healthy mucosal lining is essential for good postoperative hearing. A small middle ear cleft due to CWD surgery and presence of mucosal disease which impairs normal middle ear functions may prevent efficient ossicular chain movement and cause poor hearing outcomes in patients with an intact stapes superstructure, even if the ossicular chain is completely intact as seen in some adhesive otitis media cases.

There are controversies about the effects of the ossicular reconstruction materials on hearing outcomes, because similar hearing outcomes have been reported with different reconstruction materials [28,31,32]. By contrast, better hearing outcomes were also reported with autograft PORP compared to allograft reconstruction materials [33]. Another study reported better outcomes with titanium TORP when compared with the autologous incus [34]. In our study, no statistically significant difference could be found between the ossicular chain reconstruction materials in relation to hearing outcomes.

In conclusion, status of the middle ear mucosa and external auditory canal are important factors affecting the outcomes in cholesteatoma. Instead of a CWD surgery, a CWU surgery seems applicable in cases of cholesteatoma when the bone in the external auditory canal is not eroded by the disease.
